# The Networked Computer Metaphor: A Novel Tool for Psychiatric Trainees to Enhance Utility of the Biopsychosocial Model of Health and Illness

**DOI:** 10.7759/cureus.17395

**Published:** 2021-08-23

**Authors:** Sabish Balan, Pradipta Majumder, Rajiv Radhakrishnan, Roopma Wadhwa, Saurabh Somvanshi

**Affiliations:** 1 Psychiatry, NYC Health + Hospitals - Harlem, New York, USA; 2 Psychiatry, Drexel University College of Medicine, Philadelphia, USA; 3 Psychiatry, WellSpan Health, York, USA; 4 Psychiatry and Behavioral Sciences, Yale School of Medicine, New Haven, USA; 5 Psychiatry, South Carolina Department of Mental Health, Columbia, USA; 6 Psychiatry and Behavioral Sciences, Jamaica Hospital Medical Center, New York, USA

**Keywords:** psychiatry, psychology, psychiatric disorders, model, health, illness, psychiatry training, hardware-software-internet metaphor, networked computer metaphor, biopsychosocial model

## Abstract

The biopsychosocial (BPS) model proposed by George Engel posited that a disease developed through a complex interaction of biological, psychological and social factors. This popular model, despite its limitations, continues to influence the practice and treatment of illness and service delivery worldwide. We propose the networked computer metaphor as a novel and pragmatic tool to help psychiatric trainees appreciate and enhance the utility of the BPS model as it pertains to psychiatric disorders. We also propose that the application of this metaphor would help provide some clues to answer the question of achieving the goal envisioned by Engel of providing holistic and comprehensive patient-centered care. We also discuss the utility of this metaphor from trainee, teacher and patient perspectives and describe various examples of the application of this metaphor so as to deepen our understanding of the BPS model. We discuss the criticisms of this model, summarize the applications of this metaphor and outline future directions for research.

## Introduction

 “A metaphor is not an ornament. It is an organ of perception. Through metaphors, we see the world as one thing or another - Neil Postman”

George Engel, in 1977, proposed the biopsychosocial (BPS) model of disease that challenged the reductionism prevalent in medicine at that time and sought to bring back humanism to the field [1]. This holistic model claimed to be rooted in general systems theory and posited that an illness develops through a complex interaction of biological, psychological, and social factors. In the field of psychiatry, the BPS model as noted by Pilgrim has “become established as psychiatric orthodoxy” [2]. Ghaemi describes the model as “the status quo of contemporary psychiatry” and notes that the model has currently degenerated into a form of eclecticism where one is free to do whatever one chooses [3]. Despite various criticisms such as the model being unscientific, postmodernist/eclectic, over-inclusive and lacking in philosophical coherence to mention a few, it continues to thrive and influence the practice and treatment of illness and service-delivery throughout the world [4].

Bolton and Gillette argue in their book for a rethinking and reinvigoration of the model and note: “The answer to the content problem, we suggest, lies in scientific and clinical specifics, not generalities.” [5]

Every standard psychiatric evaluation format contains a section on BPS formulation where salient aspects of the patient’s psychiatric history are mapped onto the model’s three domains (biological, psychological, social). In this way, the evaluator is able to conceptualize the patient’s presentation in their entirety, gaining an appreciation of the complex relationships and interactions that result in the patient's final presentation of psychiatric disorder(s). Teaching BPS formulation and learning to do this well are both difficult skills [6]. However, this skill is considered a core competency by the American Board of Psychiatry and Neurology (ABPN) for practicing psychiatrists and a patient care milestone by the Accreditation Council for Graduate Medical Education (ACGME). Teaching medical students and psychiatric residents to appreciate the subtleties of this model can aid them in developing a comprehensive formulation. This can foster a deeper person-centered understanding of psychiatric illness rooted in contexts particular to each patient rather than mere attention to diagnosis of psychiatric disorder(s) and their treatment as per the current Diagnostic and Statistical Manual of Mental Disorders, 5th Edition (DSM-V) or the International Classification of Diseases, Tenth Revision (ICD-10) classification or other classificatory systems. Doing so may help to counteract some of the criticisms leveled against the reductionistic practice of psychiatry despite our best intentions and attention to the BPS model in theory [7]. The question is: Can we achieve the goal Engel envisioned 45 years after he proposed this model? We hope this paper provides some clues to answer this question.

We propose that a device such as a metaphor could provide more nuance and rethinking with a goal to improve the application of the BPS model by early psychiatric trainees with regards to diagnosis, conceptualization and treatment of psychiatric disorders. In cognitive linguistics, a conceptual metaphor refers to the understanding of one idea or conceptual domain in terms of another [8]. In the field of medicine, a metaphor is a helpful communication tool that can introduce unfamiliar material, which can be then connected to the domain of established knowledge; alternatively, the use of a metaphor can break preexisting mindset and help reframe ideas so as to make novel discoveries [9]. 

## Technical report

A metaphor that remains quite popular in our culture in this information age is that a human being, in some respects, can be conceptualized as an advanced computer or a computational machine. This idea is not new and has beginnings in the 1943 paper by McCulloch and Pitts’s [10]. They employed the tools of logic and computation developed by mathematician Alan Turing, who described “Turing Machines” to understand activities at the neural and mental levels.

**Figure 1:**
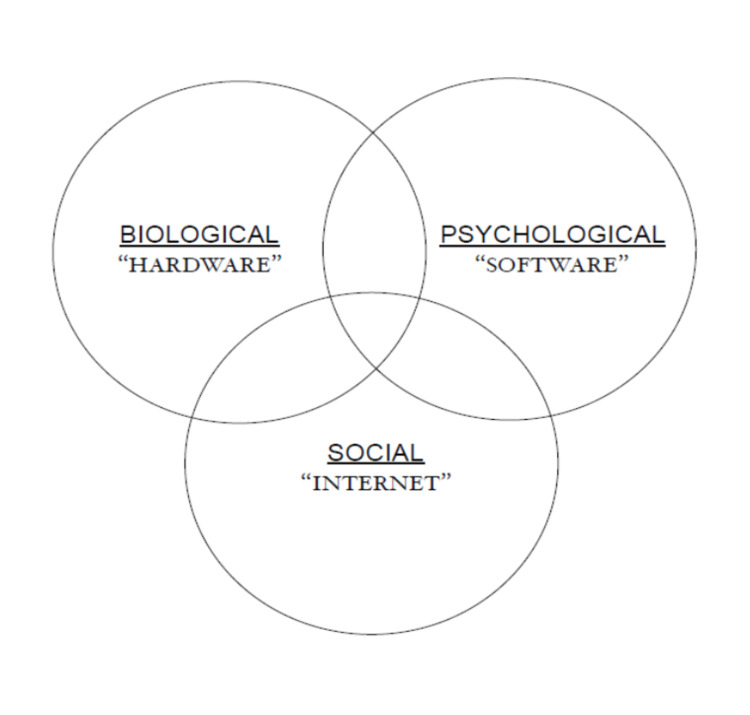
Networked computer metaphor (hardware-software-internet) mapped on to the bio-psycho-social model.

Building on the above, we arrive at the “networked computer” - a metaphor that is more intuitive and pragmatic than the aforementioned computer metaphor in terms of mapping the human being situated in their social environment. Figure 1 shows a representation of this metaphor mapped on to the BPS model. The human body in general and the brain in particular is analogous to the hardware component of the computer while the mind maps on to the computer software. Furthermore, the computer can be connected to other computers by a worldwide web or by an interconnection of networks (which in abbreviated form, is termed "internet"). The internet is analogous to the “social” element of the BPS model. One could thus posit that the optimal functioning of the computer and its applications require the best possible functioning of all these domains in an interactional framework. We could also call the networked computer metaphor, the hardware-software-internet metaphor.

On a related but parallel note, the idea of computer networks as social networks is not new and has been described by Barry Wellman, who has made immense contributions to the theory of network analysis [11]. In attempting to map the human body to the hardware in this metaphor, one needs to be cognizant that the hardware requirements to account for the complex spectrum of human behavior would entail the need for supercomputer(s) which have immensely higher performance than general purpose computers. In the same vein, while mapping the mind to software in this metaphor, one should appreciate that the software needed for performing the entire gamut of human psychological functions/operations involves complex artificial Intelligence/machine learning based software with self-regulating and self-learning algorithms [12]. In addition to this human complexity, a defining feature of us human beings is that we possess consciousness, which is the state of being aware of the self along with an awareness of the environment; understanding the role of consciousness continues to be an enigma for researchers and philosophers of science. The networked computer metaphor that we propose does not intend to suggest that human beings are automatons like computers or robots which lack consciousness - although, such questions have been increasingly raised by philosophers and artificial intelligence (AI) researchers [13].

Seeing the BPS model through the networked computer metaphor opens up exciting and novel ways of thinking about mental health and illness even though, the core metaphor is not new. The biological aspects of mental health and illness, to name a few, includes various factors such as age, sex, family history, medical history, developmental history, genetics and epigenetic modifications, nature of foods ingested (e.g., composition of essential nutrients in foods) and recreational drug use; all these listed factors, under specific conditions, can interact and lead to the onset and maintenance of mental illness. From this networked computer perspective, dysfunction in the biological aspects is akin to the “hardware” malfunction that could even shut down the biological "machinery". One can use the "hardware" analogy to discuss about subtle aspects of cognition such as information processing deficits noted in illnesses such as schizophrenia and propose “hardware” solutions to patients. This could include measures such as engaging in dietary modifications, pharmacotherapy and other somatic treatments [such as electroconvulsive therapy (ECT), transcranial magnetic stimulation (TMS) and deep brain stimulation (DBS)]. In using this metaphor as an aid to educating patients, there is an added potential to reduce nonadherence to proposed biological treatments given its explanatory potential.

Another dimension of the BPS model is the realm of Psychology. Psychology, by definition, is the study of mind and behavior as per the American Psychological Association (APA) definition ( [14]. The APA dictionary of psychology defines the mind in one way as “broadly all intellectual and psychological phenomena of an organism, encompassing motivational, affective, behavioral, perceptual, and cognitive systems; that is, the organized totality of an organism’s mental and psychic processes and the structural and functional cognitive components on which they depend… The term, however, is also used more narrowly to denote only cognitive activities and functions, such as perceiving, attending, thinking, problem solving, language, learning, and memory” [14]. The psychological aspects of us, to name a few, include the functions such as the thoughts, feelings, emotions, beliefs, attitudes, personality attributes, defense mechanisms, ego functions and coping styles. All these aspects of the mind help us as humans to operate in the world analogous to the “software” aspect of the computer (especially the “operating system” aspect of the software). Using this "software/ operating system" analogy for the psychological domain, one can postulate, for example, that patients with personality disorders use different/aberrant/primitive/maladaptive operating systems that have not undergone age-appropriate "upgrades" or modifications, possibly as a result of early attachment disruptions, deprivations and/or trauma. The networked metaphor would help psychiatric trainees identify those patients who need psychotherapy, educate them to seek psychotherapy (if indicated) and further, advocate need for psychotherapeutic interventions more vociferously when dealing with ancillary agencies such as insurance companies. Furthermore, this metaphor may allow room for discussion and further psychoeducation when patients refuse psychotherapy to help in engagement. In addition, one could emphasize to patients that their “software” related problems (related to psychological issues) require “software” related solutions and that application of “hardware” solutions (such as medication management) alone may not be sufficient. If "software" solutions are left untreated as in patients who refuse psychological interventions, this neglect can continue to perpetuate the psychiatric illness. We could further postulate that the more “insight-oriented” psychotherapeutic treatments would involve an understanding of the deeper “software” aspects akin to the “operating system." In contrast, cognitive-behavioral therapy (CBT) or dialectical behavior therapy (DBT) involve primarily, focus on learning new skills which are similar to that of installing new “apps” (applications) on the computer that help with certain applications (“provision of adaptive coping skills”). Seen in this way, the trainees have more dexterity in providing psychoeducation to patients.

In turning our attention to social factors, we note that social connections to name a few, include relationships such as family/ social/ peer support networks and those networks related to professional, religious and cultural aspects. We know, from a review of the literature, that lack of social networks (and consequent isolation) or the presence of dysfunctional networks (that lead to stress such as adverse childhood experiences and exposure to violence), can increase depression [15] and cause early mortality [16]. Developmentally, there is genetic predisposition for infants to connect with care givers who become “attachment figures" as child grows. This becomes the template for further adult relationships. Given this propensity of being hardwired to connect with other people, developing healthy social connections thus becomes integral for one’s wellbeing. We could educate patients and encourage them to make prosocial connections more effectively using this networked computer metaphor. One could liken our individual isolated self akin to that of an "offline" computer that possesses hardware and software components but lacks an "internet connection" which would enable it to be connected to other computers (which, in the case of humans would imply, connections with other people). In the absence of an "internet connection", reciprocal communication and relationships with others are not possible. This causes social isolation, a feature that we see in humans as commonly related to the onset of depression.

Thinking further, the proposed metaphor could also help us engage patients who have difficulties developing social connections and one could further encourage them to learn social skills. Building upon this, we could explain to such patients that many dysfunctional networks in which they engage cause socio-occupational dysfunction, adversely affecting their mental and physical health (with short-term payoffs like euphoria and long-term adverse health consequences). These dysfunctional networks, for example, include networks such as drug-using relationships between people (where social networks are predicated on the use of recreational drugs). When seen from the networked computer metaphor standpoint, they are akin to "unsecure" networks (whereby, maladaptive/manipulative "programs" like "viruses" can enter the computer and destroy the hardware and software) and this can cause patients to sabotage their sobriety (as in those with Addictive Disorders). These dysfunctional networks could be replaced by more “secure” networks, that are conducive to psychiatric recovery like the 12-step groups [such as Alcoholic Anonymous (AA) and Narcotic Anonymous (NA) for alcohol use disorder and opioid use disorder, respectively] or other group psychotherapy networks. These networks afford more healthy and reciprocal interactions predicated on maintaining the patient’s sobriety as in the case of substance use disorders and promote illness recovery.

Application of this metaphor could be a good tool for academic psychiatrists to teach the BPS model to trainees in general and BPS formulation in particular. This would help reduce stigma from a patient perspective for psychiatric disorders given that traditionally, these disorders have been regarded as “mental illnesses” related to problems in the “mind"; psychiatric disorders can now be seen from a broader biopsychosocial model just as is seen in other chronic medical disorders such as diabetes and hypertension. From a clinician's perspective, use of this metaphor could have positive ramifications in terms of developing and better operationalizing individualized treatment plans based on current evidence such as the use of particular medications or psychotherapies and provision of healthy social support systems and entitlements depending on the patient's needs. This can improve adherence to treatment plans and reduce medication side effects while simultaneously addressing core problem/s as opposed to resorting to medication treatments reflexively and paying little attention to psychosocial problems. Exploring a person’s burden of psychiatric illness through the networked computer metaphor could thus limit biological reductionism and allow for a more holistic and integrative approach. The persistence of biological hegemony, attributed to being under the grip of the mainstream neuroscience ideology in our mental health landscape, has led to a lack of insurance parity for psychiatric services that are not deemed “biological”. Consequently, this practice has resulted in low insurance reimbursement rates for psychological and social treatments. The networked computer metaphor might help identify and reduce the biological bias and help providers become more attuned to the psychological and social perspectives in the treatment of psychiatric illness.

## Discussion

The challenge with the biopsychosocial model is that it does not intuitively guide students/trainees to choose the appropriate modality of treatment (pharmacological/psychological/social) or their combination; nor does it help patients appreciate why a particular treatment modality was recommended. However, the networked computer metaphor allows an intuitive understanding for a novice student/trainee/patient with limited understanding of biological/psychological/social processes (based on mere basic familiarity with how networked computers operate) of the difficulties that may arise (in terms of hardware vs. software vs. internet issues) and how they can be corrected. Another advantage of this metaphor is that we can build upon the networked metaphor with some creativity to map on to additional perspectives to extend the basic BPS model to include bio-psycho-social-existential and bio-psycho-social-spiritual models which could also be mapped on to specific aspects of the software.

The “computer metaphor” is not, however, without its critics; the same criticisms could also be levelled against the networked computer metaphor. The eminent mathematician George E.P. Box put it cogently that "All models are wrong, but some are useful". Like Freud’s early hydraulic model, every age of psychiatry, in part to achieve scientific gravitas, has offered models of the mind metaphorically built on historically contemporaneous models in physics. In social science, most models are metaphors in that finding causal scientific laws can be complicated/difficult/impossible and we have to resort to use of pragmatic models that have practical utility and less validity as an alternative. It is these models that underlie the basis of our present classificatory systems such as the ICD, DSM and RDoC for psychiatric disorders. Kendler has criticized the dualistic “either/or thinking” prevalent in psychiatry as a result of three causes namely Cartesianism (physical versus mental disorders), nineteenth-century neuropathology (organic versus functional disorders), and computer functionalism (hardware versus software) and he argues for an empirically based pluralism [17]. At the same time, one cannot overlook the fact that the mind-body problem remains an enigma in Western philosophy/science and this has implications for psychiatric nosology and research. We do not aim to foster this dualistic either/or thinking through the use of the networked computer metaphor but argue for a "yes and" improvisational thinking which allows for an appreciation that aberrations in one or more domains can result in complex effects in other domains [18]. From a complex systems perspective, human processes and interactions follow nonlinear dynamics which are chaotic and the use of networked computer metaphor may foster this appreciation of nonlinear dynamic systems where effects in one domain can produce unpredictable effects in other domains [19]. One of the limitations that we foresee is that the applicability of this metaphor may be restricted to areas of the world where educators, trainees and patients are familiar with computers. Here again, one could improvise this networked computer metaphor and rename it as “smart phone” metaphor, which is essentially, a networked computer; this metaphor would then be applicable in those geographic locations.

McHugh and Slavney noted that the analogy of the BPS model is like a list of ingredients as opposed to a recipe [20]. They go on to say that we need more than mere names of ingredients to cook a meal, and that a recipe includes the quantity of the ingredients as well as the order in which the ingredients are added. From our experience teaching medical students and residents, we note that many of the psychiatry trainees get stuck at the level of “naming” the ingredients and have difficulty getting to appreciate the “quantity” of ingredients, “order” of addition, and thinking about how the recipe with these ingredients dynamically resulted in the “preparation” of the final meal (which for the patient would be the manifestations of the psychiatric disorder they present with). We hope that mapping the networked computer metaphor onto the BPS model may help to put weights on the “ingredients” required to "cook" an individual "meal". This will also help delineate, hypothesize, and “reverse engineer” the order in which the "ingredients" and their addition may have played a causal role in the preparation of the "meal". This metaphor may thus help articulate ways to operationalize and increase clinical utility and scientific specificity of the BPS model.

## Conclusions

In summary, the use of networked computer metaphor could be a valuable tool for medical students, psychiatry residents, academic psychiatrists to appreciate the nuances of the BPS model of psychiatric illness and improve its applicability in regards to formulation, diagnosis and treatment. Application of this metaphor would help to achieve the vision of Engel when he first proposed the BPS model with the goal of providing comprehensive patient-centered care. This would be a good tool for academic psychiatrists teaching psychiatric trainees about the BPS model in general and BPS formulation in particular. However, it must be noted that it is not our intention in any way while proposing this metaphor to cause further reification or make the cleavage between the biological, psychological and social domains any deeper than the original model proposed by Engel, who intended it to be comprehensive. At the clinical trenches where care is provided within the constraints of time, we hope that this metaphor will be a good tool to provide efficient and comprehensive care. Even as we write this paper, we are cognizant of many parts of the world where psychiatric services are solely limited to the provision of medications with a lack of attention to the psychosocial needs of patients. The proposed metaphor could rectify this skewing that favors excessive use of biological interventions. As a specialty, we could attract more medical students to the pursuit of psychiatry as they appreciate the various dimensions of this model and see psychiatry as a holistic discipline that pays specific attention to its biological, psychological and social dimensions. Moreover, it could reduce the stigma that many of our patient’s experience that results in delays in care and societal marginalization. We could also test hypotheses empirically to study the impact of the networked computer metaphor on enhancing our knowledge of psychiatric disorders to generate better and comprehensive treatment outcomes. In addition, this could also have applications in fields outside of psychiatry such as medicine, nursing, social work and psychology.
